# Estimating the epidemic size of chikungunya virus infection in Guangzhou, China, from July to September 2025: a single-center cross-sectional study

**DOI:** 10.1080/22221751.2026.2645833

**Published:** 2026-03-30

**Authors:** Weiguo Lu, Zihao Guo, Ka Chun Chong, Junyuan Huang, Chunke Chen, David S Hui, Shi Zhao, Chris Ka Pun Mok

**Affiliations:** a First Affiliated Hospital of Guangzhou University of Chinese Medicine, Guangzhou, Guangdong, People’s Republic of China; b Jockey Club School of Public Health and Primary Care, The Chinese University of Hong Kong, Hong Kong, Hong Kong SAR, China; c Centre for Health Systems and Policy Research, Faculty of Medicine, The Chinese University of Hong Kong, Shatin, Hong Kong SAR, China; d Li Ka Shing Institute of Health Sciences, Faculty of Medicine, The Chinese University of Hong Kong, Hong Kong, Hong Kong SAR, China; e SH Ho Research Centre for Emerging Infectious Diseases, Faculty of Medicine, The Chinese University of Hong Kong, Hong Kong, Hong Kong SAR, China; f Department of Medicine & Therapeutics, The Chinese University of Hong, Hong Kong, Hong Kong SAR, China; g School of Public Health, Tianjin Medical University, Tianjin, People’s Republic of China; h School of Biomedical Sciences, The Chinese University of Hong Kong, Hong Kong, Hong Kong SAR, China

**Keywords:** Chikungunya, attack rate, seroprevalence

## Abstract

Although no previous large-scale outbreak was reported in China in recent years, since July 2025, a surge in the number of chikungunya virus infections has been observed in multiple cities of Guangdong province following the introduction of imported cases, including Guangzhou. Using serological and epidemiological data obtained during a chikungunya outbreak in Guangzhou city, we estimated an attack rate of 22% for chikungunya virus infection. Accounting for case under-reporting is crucial for accurate disease burden assessment and planning of future vaccination programmes.

## Introduction

In recent years, there has been a significant increase in chikungunya virus (CHIKV) infections reported in an expanding range of geographical regions globally [1]. Since July 2025, Guangdong province, China, experienced unprecedented outbreaks of chikungunya, with more than 25,000 confirmed CHIKV infections reported as of November 8 [2]. Guangzhou, the capital of Guangdong province, has a humid subtropical climate characterized by abundant rainfall and high temperatures from April to September. These conditions favour the breeding of *Aedes albopictus*, the primary vector for CHIKV and dengue virus. Although dengue fever cases occurred annually in Guangzhou, local CHIKV infections were historically rare, with only imported cases with international travel history reported between 2017 and 2019 [3]. Following the confirmation of Guangzhou's first CHIKV infection on July 8, 2025, a series of vector-control measures were conducted throughout the city, including a month-long, community-wide mosquito extermination campaign [4,5]. As of September 23, a total of 599 cases (3.16 cases per 100,000 population) were reported, and the CHIKV epidemic had almost ceased at the community level in Guangzhou by November 2025 [2].

However, CHIKV surveillance primarily captured symptomatic, laboratory-confirmed cases from public and private healthcare facilities. Substantial under-reporting is therefore expected [6], especially given the high rate of asymptomatic infections [7]. This highlights a critical need for accurately quantifying the true infection burden to inform effective vaccination strategies for future vaccine campaigns. In this study, we used serological data to estimate the epidemic size in a CHIKV-naive population during the CHIKV outbreak in Guangzhou [8].

## Methods

We collected serum samples from 2256 healthy individuals (age range: 2–90 years; mean [SD] age: 42.5 [14.8]) who underwent health screening and have no history of CHIKV infection based on self-report at the First Affiliated Hospital of Guangzhou University of Chinese Medicine, Guangzhou, China, from July 21 to September 9, 2025. The First Affiliated Hospital of Guangzhou University of Chinese Medicine is a tertiary hospital located in the urban area of Guangzhou city. Our study was approved by the human ethics committee of the First Affiliated Hospital of Guangzhou University of Chinese Medicine (approval no: K-2025-133). All serum samples were laboratory tested for chikungunya virus antibodies by enzyme-linked immunosorbent assay (ELISA) (supplementary materials S1). The daily number of reported CHIKV infections was retrieved from the National Notifiable Infectious Disease Reporting System (NNDRS) of China. According to the Chikungunya Fever Prevention and Control Protocol (2025 Edition), all medical institutions in China are mandated to report all suspected, probable, and confirmed CHIKV patients to the NNDRS within 24 h of case ascertainment [9].

By coupling the data on observed seropositive rate and reported infections over the course of the outbreak with an epidemic model describing the transmission process of the disease, we estimated the infection attack rate (IAR) and time-varying reproduction number (*R_t_*) of CHIKV infection. These estimates accounted for infection under-reporting, the sensitivity and specificity of the laboratory test, and the presence of imported cases [10]. In our study, imported cases were identified based on epidemiological investigation on the recent travel history, as well as a linked exposure history of CHIKV. We also estimated *R_t_* using reported infections alone for comparison. We assumed 99.99% of the population in Guangzhou were susceptible of CHIKV infection, providing no large-scale outbreak reported previously [3]. We estimated the model parameters through a Bayesian statistical framework using the Markov chain Monte Carlo (MCMC) with non-informative prior distributions. We obtained the median value and 95% credible intervals (CrIs) from the posterior distributions. The methodology of the study was detailed in supplementary materials S2.

## Results and discussion

Of the 2256 study participants who have no self-report history of CHIKV infection, 932 (41.3%) were female and 1324 (58.7%) were male, with an overall seropositive rate of 19.6%, which aligns with a pooled seroprevalence estimate of 24% from previous population-based serosurveys of asymptomatic individuals [11]. The seropositive rate was significantly higher in males than in females (23.0% versus 14.8%, *p*-value < 0.001), but no evident difference across age groups (Table S1).

**Figure 1.**
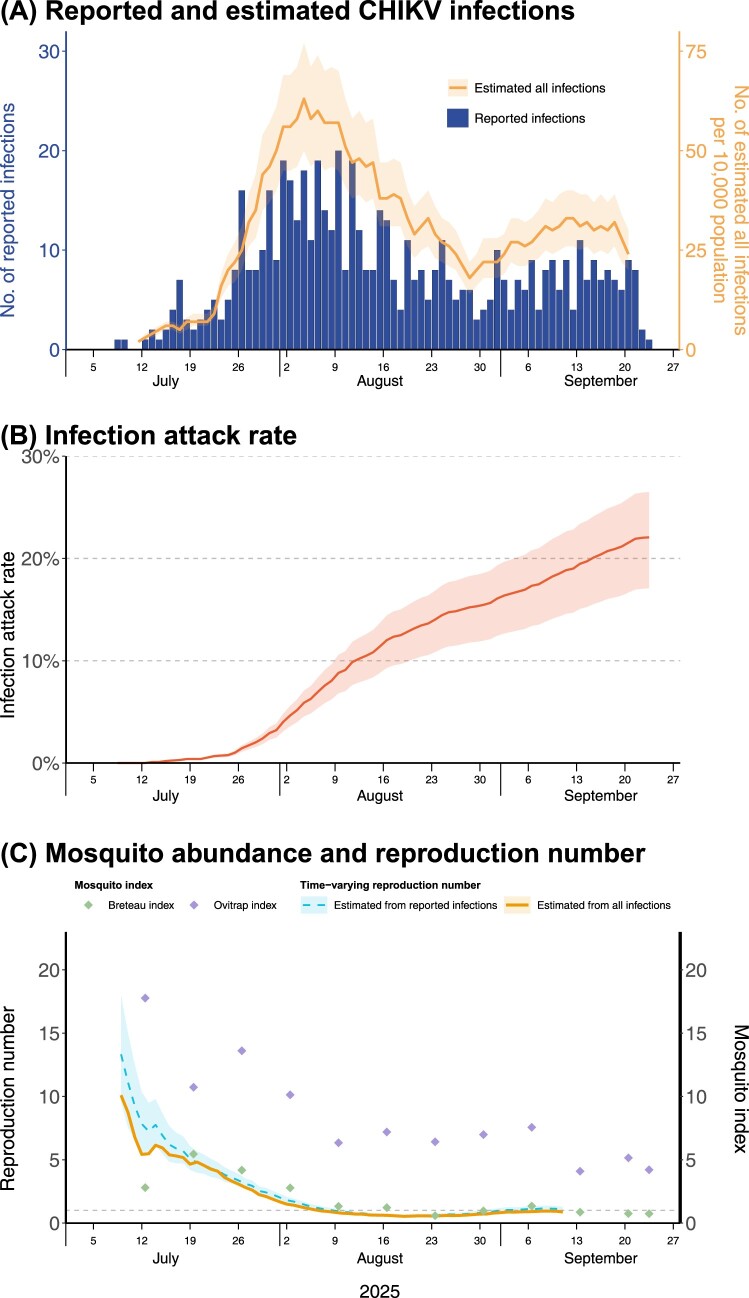
Reported and estimated CHIKV infections and transmission risks in Guangzhou, China, from July 8 to September 23, 2025. In panel (A), blue bars represent daily number of reported CHIKV infections, and yellow solid line represent 7-day moving average of the median CHIKV infections estimates, with shaded region representing the 95% credible interval. The right y-axis is scaled based on the population size of Guangzhou. Panel (B) showed estimated infection attack rate through time. The solid line represents the median estimates and the shaded region represents the 95% credible interval. Panel (C) showed estimated *R_t_* values and observed level of mosquito abundance. Solid lines represent median estimates from reported infections (blue) and all infections (yellow), and shaded regions represent 95% credible interval. The diamond points represent the Breteau index (green) and Ovitrap index (purple). The horizontal dashed line marks the reproduction number threshold of 1. In all panels, the ticks on the x-axis mark the epidemiological weeks in 2025.

From the epidemic model, we jointly estimated the time-varying *R_t_* and IAR of CHIKV. We found that a significant proportion of CHIKV infections in Guangzhou were under-reported (Figure 1A), with a reporting ratio of 0.77% (95% CrI: 0.68–0.95) among all symptomatic CHIKV infections. By the end of the study period on September 23, 2025, the IAR for CHIKV epidemic in Guangzhou was estimated at 22.0% (95% CrI: 17.1–26.5) (Figure 1B). The estimated *R_t_* values were consistently lower when accounting for under-reporting than the unadjusted *R_t_* estimates from only reported cases (Figure 1C), which avoided the overestimation of transmission risks. The initial *R_t_* was 9.89 (95% CrI: 9.83–9.95) during the first epidemic week of the CHIKV outbreak. According to the vector surveillance data in Guangzhou city [11], mosquito abundance (as measured by Breteau index and Ovitrap index) steadily decreased over time. This trend was strongly consistent with the decline in *R_t_* estimates (Pearson’s correlation coefficient = 0.95 [Breteau index] and 0.93 [Ovitrap index]; both *p*-values < 0.001), suggesting a likely effectiveness of local mosquito control measures in controlling the CHIKV outbreak (Figure 1C).

Previous seroprevalence estimates for CHIKV infection were primarily derived from asymptomatic populations [1,12]. By integrating reported symptomatic case data with an epidemic model, we inferred an CHIKV IAR of 22% for the 2025 Guangzhou outbreak. This estimate falls within the range (from 10.2% to 63%) reported by serological surveys of historical CHIKV outbreaks in general populations across various regions (supplementary materials S3). Given that CHIKV infection would confer long-lasting immunity, our estimated IAR, combined with an estimated basic reproduction number (*R*_0_) for CHIKV, can provide a clear target for vaccination, should a chikungunya vaccine become available. By calculating the herd immunity threshold from *R*_0_, the remaining proportion of the population requiring vaccination can be directly inferred from our findings. This would be particularly informative for public health preparedness and for mounting an early response to prevent a large-scale outbreak. Furthermore, the observed low case reporting rate suggested that a substantial proportion of CHIKV infections were asymptomatic. Therefore, it is imperative to closely monitor transmission risk throughout a CHIKV outbreak, which enables timely implementation of control measures and transparent public communication about infection risks, thereby helping to prevent widespread transmission.

This study had several limitations. First, the serum samples were collected from individuals who underwent health screening at a single healthcare facility. Selection bias may exist regarding the representativeness of the study population to the general population in Guangzhou. The age structure of the study samples (age structure: 0–14 years: 0.7%, 15–59 years: 86.1%, ≥ 60 years: 13.2%) skewed to adult population compared to that of the general population (0–14 years: 13.9%, 15–59 years: 74.7%, ≥ 60 years: 11.4%). The sample also contained a higher proportion of males (58.7%) compared to the general population (52.8%). Such discrepancies could be attributed to the difference in health-seeking behaviour among the population groups. Given the observed seropositive rate is higher in males than in females, the infection attack rate could be overestimated to some extent. Population-based serological survey is warranted to confirm our study findings. Second, the population at risk of CHIKV infection may be smaller than what we assumed in the current study, given the heterogeneous mosquito distribution across geographic areas of the city [5]. Third, a precise estimate of the asymptomatic ratio is not feasible as the serum samples might be from infected individuals who were at pre-symptomatic stage. Last, although we collected serological data from a tertiary hospital in the urban area in Guangzhou, selection bias cannot be ruled out. Individuals residing in rural areas may have different healthcare-seeking behaviours and exposure risks compared to their urban counterparts. Therefore, our findings may not be fully representative of the entire Guangzhou population, and the results should be interpreted with caution.

## Conclusions

Despite substantial geographic heterogeneity in attack rates observed among these studies, our findings add evidence on the disease burden of CHIKV in a densely populated subtropical region. Our findings suggest that only a small fraction of CHIKV infections were reported during the peaking period of 2025 outbreak in Guangzhou, China. Given the estimated high attack rate of the ongoing CHIKV epidemic, reliance on passive disease surveillance alone appears insufficient for reflecting the real-time risk of CHIKV transmission.

## Supplementary Material

Supplemental Material
