# Supplementary material for: Estimating the epidemic size of chikungunya virus infection in Guangzhou, China, from July to September 2025: a single-center cross-sectional study
Source: Emerg Microbes Infect. 2026 Mar 30;15(1):2645833. doi: 10.1080/22221751.2026.2645833 (PMC13037205; doi:10.1080/22221751.2026.2645833)
Supplement: Supplemental Material [file TEMI_A_2645833_SM2299.docx]

**Supplementary materials**

Table of Contents

[S1 Laboratory methods 2](#_Toc223295423)

[*S1.1 Specimen collection* 2](#_Toc223295424)

[*S1.2 ELISA binding assay* 2](#_Toc223295425)

[S2 Statistical framework 3](#_Toc223295426)

[*S2.1 Epidemic model* 3](#_Toc223295427)

[*S2.2 Joint likelihood framework and parameter estimation* 4](#_Toc223295428)

[S3 Summary of infection attack rate estimates in historical CHIKV outbreaks 5](#_Toc223295429)

[Table S1.. 6](#_Toc223295430)

[Table S2. 7](#_Toc223295431)

[Figure S1 8](#_Toc223295432)

[Figure S2. 9](#_Toc223295433)

[Supplementary references 10](#_Toc223295434)

# **S1 Laboratory methods**

## *S1.1 Specimen collection*

Specimens of clotted blood were collected from the patients. The serum was separated from the blood by centrifugation and stored at -80 until use. This study was approved by the human ethics committee of the First Affiliated Hospital of Guangzhou University of Chinese Medicine (Approval no. K2025-133).

## *S1.2 ELISA binding assay*

96-well enzyme-linked immunosorbent assay (ELISA) plates (Nunc MaxiSorp, Thermo Fisher Scientific) were first coated overnight with 100ng per well of purified recombinant envelope 2 protein of Chikungunya virus (Sino Biological) in PBS buffer. The plates coated with the purified recombinant protein were then blocked with 100ul of Chonblock Blocking/Sample Dilution ELISA Buffer (Chondrex, Inc, USA) at room temperature for 2 hours. Each serum or plasma sample was tested at a dilution of 1:100 in Chonblock Blocking/Sample Dilution ELISA Buffer and 100ul of diluted sample was added to the ELISA wells of each plate for 2-hour incubation at 37°C. After extensive washing with PBS containing 0.1% Tween 20, HRP-conjugated goat anti-human IgG (1:5000, GE Healthcare) was added for 1 hour at 37°C. The ELISA plates were then washed five times with PBS containing 0.1% Tween 20. Subsequently, 100 μL of HRP substrate (Ncm TMB One) (New Cell & Molecular Biotech Co. Ltd, China) was added into each well. After 15 minutes incubation, the reaction was stopped by adding 50 μL of 2 M H2SO4 solution and analyzed on an absorbance microplate reader at 450 nm wavelength.

The assay was initially validated using 100 negative controls from adults in Hong Kong. We defined a serum to be positive if the OD value was one standard deviations (SD) above the mean of the negative controls, which in our assay was 0.358.

# **S2 Statistical framework**

## *S2.1 Epidemic model*

Among all individuals who were infected by chikungunya virus (CHIKV) in Guangzhou city, China, we consider them to be categorized into 2 mutually exclusive and collectively exhaustive groups (i.e., groups 1 and 2), including

- group 1: symptomatic infections (with symptomatic ratio of $1-\alpha$):
  - test-positive and reported (with reporting ratio of $\theta$),
  - never tested, and thus unreported (with proportion of $1-\theta$), and
- group 2: asymptomatic infections (with asymptomatic ratio of $\alpha$), and in this study, all asymptomatic infections were not reported.

We assumed the observed daily number of reported infections ($Z_{t}$) were drawn from a Poisson process, which is a common method to account for the systematic noise of the observational process of infections [1]:

$Z_{t} \sim\mathrm{Poisson}(\mathrm{rate}=\lambda_{t})$.

Here, $\lambda_{t}$ is the expected number of reported infections at day *t*, which was generated from the epidemic model. By using the renewal process model with consideration on the difference in transmission risks of imported and local infections [2], the transmission model can be formulated as

$\lambda_{t}=R_{t}\sum_{\tau=1}^{t} \left[ (bY_{t-\tau}^{\mathrm{import}}+C_{t-\tau})w_{\tau} \right]$, where

- $R_{t}$ is the time-varying reproduction number on day *t*,
- $Y^{\mathrm{import}}$ denotes the daily number of imported infections (Figure S1),
- $C_{t-\tau}$ denotes the daily number of local infections on day ($t-\tau$),
- $w_{\tau}$ is the serial interval distribution [3], and further discretized with step-length to be 1 day. Thus, $w_{\tau}$ denotes the probability mass value of the serial interval on day *t* after the onset of source case. We considered $w_{\tau}$ as a discretized gamma distribution with a mean of 14 days, and a standard deviation (SD) of 6.4 days [4].
- $b$ is a scaling factor considering a weakened transmissibility of imported infections compared with the local infections [2].

We remarked that the main results in this study appeared largely insensitive to a mild variation of $b$ value.

Then, we considered $\lambda_{t}$ followed a binomial process to account for a random sampling process of chikungunya case ascertainment from the total number of CHIKV infections at day *t*:

$\lambda_{t} \sim\mathrm{Binomial}\left( size=C_{t},\mathrm{probability}=\theta(1-\alpha) \right)$.

Here, $C_{t}$ is the daily number of total local CHIKV infections (i.e., including both symptomatic and asymptomatic infections). In addition, $\theta$ is the reporting ratio of among symptomatic infections (i.e., in group 1), and $\alpha$ is the asymptomatic ratio (i.e., in group 2), where all asymptomatic infections were not reported. Therefore, $\theta(1-\alpha)$ is the overall reporting ratio of the total infections (i.e., including both symptomatic and asymptomatic infections).

Similarly, we assumed the observed number of seropositive samples ($M_{t}$) at day *t* were drawn from a hypergeometric process [5]:

$M_{t} \sim\mathrm{hypergeom}\left( total size=N\cdot\left[ S_{0}-(1-\alpha)\theta\cdot\mathrm{IAR}_{t} \right],test positive=N\cdot\left[ (1-\alpha)(1-\theta)+\alpha\right]T_{\mathrm{sen}}\cdot\mathrm{IAR}_{t},K_{t} \right)$.

The probability model here describes that on the *t*-th day,

- the size of population never reported for CHIKV infection is $N\cdot\left[ S_{0}-(1-\alpha)\theta\cdot\mathrm{IAR}_{t} \right]$, where
  - $N$ is the population size of Guangzhou city, China, and we set $N$ as 18.9 million,
  - $S_{0}$ is the proportion of susceptible population before the CHIKV outbreak, and we set $S_{0}$ as 99.99%, considering that no large-scale CHIKV outbreak was reported previously in Guangdong province, China [4],
  - $\mathrm{IAR}_{t}$ is the infections attack rate, which is expressed as $\frac{\sum_{0}^{t} C_{t}}{N}$,
- the cumulative number of CHIKV infections who would be test-positive (but remained untested) for CHIKV infection is $N\cdot\left[ (1-\alpha)(1-\theta)+\alpha\right]T_{\mathrm{sen}}\cdot\mathrm{IAR}_{t}$, where
  - $T_{\mathrm{sen}}$ is the testing sensitivity of the ELISA test used for CHIKV infection history in this study, which is fixed at 75%,
  - in addition, we also set the testing specificity 100%, and
- $K_{t}$ is the sample size for serum collection and tested for CHIKV infections history in this study.

Changing those fixed values of model parameters would not affect the modelling results.

A visual presentation of the data structure and modelling framework is detailed in Figure S2.

## *S2.2 Joint likelihood framework and parameter estimation*

The joint likelihood of observing a given number of reported cases ($Z_{t}$) and seropositive samples ($M_{t}$) at day *t* over the whole study period (*T*), from July 8 to September 23, 2025, is given by:

$P\left( Z_{t},M_{t} | \theta,\alpha,R_{t} \right)=\prod_{t=\tau}^{T} \left[ P\left( Z_{t} | Z_{t-\tau+1},\ldots,Z_{t-1}, R_{t} \right)\cdot P\left( \lambda_{t} | C_{t},\theta\right) \right]\cdot P\left( M_{t} | C_{t},\alpha,K_{t} \right)$.

We jointly estimated the $\theta$, $\alpha$ and $R_{t}$ in a sliding window process with a window length ($\tau$) equals to 14 days.

A Bayesian statistical framework by using the Markov chain Monte Carlo (MCMC) method with a non-informative prior distribution (i.e., uniform distribution). Marginal posterior distributions were obtained from 4 MCMC chains with 140000 iterations for each chain, among which the first 40000 samples were discarded as for burn-in. The convergence of each MCMC chain was checked by using the trace plot, and Gelman-Rubin-Brooks convergence diagnostic [7]. We inferred the $\mathrm{IAR}_{t}$ based on the posterior distributions of $\theta$ and $\alpha$. For comparison, the time-varying reproduction number was also estimated by using the daily reported cases alone. The median as well as 95% credible interval of posterior samples were summarized.

# **S3 Summary of infection attack rate estimates in historical CHIKV outbreaks**

The infection attack rate estimated from previous serosurveys conducted in general populations of different regions is summarized in Table S2. Studies only presented results obtained from asymptomatic populations were not included. The infection attack rate from the included studies varied from 10.2% to 63%.

# **Table S1**. Demographic characteristics of study participants and summary of laboratory test results.

|  | Positive sample, *n* (%) | Total sample, *n* (%) | Positive rate, % | p value^†^ |
| --- | --- | --- | --- | --- |
| Overall | 442 (100.0) | 2256 (100.0) | 19.6 |  |
| Sex |  |  |  | < 0.001 |
| Female | 138 (31.2) | 932 (41.3) | 14.8 |  |
| Male | 304 (68.8) | 1324 (58.7) | 23.0 |  |
| Age group |  |  |  | 0.093 |
| ≤ 14 yrs | 2 (0.5) | 15 (0.6) | 13.3 |  |
| 15–60 yrs | 368 (83.3) | 1943 (86.1) | 18.9 |  |
| ≥ 60 yrs | 72 (16.2) | 298 (13.3) | 24.2 |  |

*^†^ P values were calculated by 2-sided Fisher’s exact test.*

Table S2. Historical chikungunya serosurveys among general population^#^.

| **Region** | **Study design** | **Sample size** | **Year of the survey** | **Infection attack rate (%)** | **Reference** |
| --- | --- | --- | --- | --- | --- |
| Emilia-Romagna Region, Italy | Cross-sectional | 325 | 2007 | 10.2 | [8] |
| Mandalay, Yangon, and Myeik, Myanmar | Cross-sectional | 1544 | 2013, 2015, 2018 | 34.5 | [9] |
| Mayotte, France | Cross-sectional | 1154 | 2006 | 37.2 | [10] |
| La Réunion Island, France | Cross-sectional | 2442 | 2006 | 38.2 | [11] |
| Bagan Panchor, Malaysia | Cross-sectional | 180 | 2007 | 55.6 | [12] |
| Grande Comore Island, Union of the Comoros | Cross-sectional | 481 | 2017 | 63.0 | [13] |

***^#^****Including symptomatic infections, asymptomatic infections, and healthy individuals.*

******Figure S1.**

Daily number of reported infections in Guangzhou from July to September 2025. Imported infections (yellow bar) and local infections (blue bar).

# **Figure S2.**

Visual presentation of the data structure and modelling framework. $S_{0}$=99.99%. $T_{\mathrm{sen}}$=75%. The yellow box represent the estimand; blue boxes represent observed data.

#

# **Supplementary references**

1. Cori A, Ferguson NM, Fraser C, Cauchemez S. A new framework and software to estimate time-varying reproduction numbers during epidemics. Am J Epidemiol. 2013;178(9):1505–12.

2. Thompson RN, Stockwin JE, van Gaalen RD, Polonsky JA, Kamvar ZN, Demarsh PA, et al. Improved inference of time-varying reproduction numbers during infectious disease outbreaks. Epidemics. 2019;29(100356):100356.

3. Fine PEM. The interval between successive cases of an infectious disease. Am J Epidemiol. 2003;158(11):1039–47.

4. Zhang M, Li Y, Huang X, Liu M, Jiang S, Zeng B, et al. Epidemiological characteristics and transmission dynamics of the early stage Chikungunya fever outbreak in Foshan City, Guangdong Province, China in 2025. Infect Dis Poverty. 2025;14(1):93.

5. Zhao S, Mok CKP, Tang YS, Chen C, Sun Y, Chong KC, et al. Inferring incidence of unreported SARS-CoV-2 infections using seroprevalence of open reading frame 8 antigen, Hong Kong. Emerg Infect Dis. 2024;30(2):325–8.

6. Gelman A, Carlin JB, Stern HS, Dunson DB, Vehtari A, Rubin DB. Bayesian data analysis, third edition. 3rd ed. Philadelphia, PA: Chapman & Hall/CRC; 2013.

7. Ribeiro Dos Santos G, Jawed F, Mukandavire C, Deol A, Scarponi D, Mboera LEG, et al. Global burden of chikungunya virus infections and the potential benefit of vaccination campaigns. Nat Med. 2025;31(7):2342–9.

8. Moro ML, Gagliotti C, Silvi G, Angelini R, Sambri V, Rezza G, et al. Chikungunya virus in North-Eastern Italy: a seroprevalence survey. Am J Trop Med Hyg. 2010;82(3):508–11.

9. Luvai EAC, Kyaw AK, Sabin NS, Yu F, Hmone SW, Thant KZ, et al. Evidence of Chikungunya virus seroprevalence in Myanmar among dengue-suspected patients and healthy volunteers in 2013, 2015, and 2018. PLoS Negl Trop Dis. 2021;15(12):e0009961.

10. Sissoko D, Moendandze A, Malvy D, Giry C, Ezzedine K, Solet JL, et al. Seroprevalence and risk factors of chikungunya virus infection in Mayotte, Indian Ocean, 2005-2006: a population-based survey. PLoS One. 2008;3(8):e3066.

11. Gérardin P, Guernier V, Perrau J, Fianu A, Le Roux K, Grivard P, et al. Estimating Chikungunya prevalence in La Réunion Island outbreak by serosurveys: two methods for two critical times of the epidemic. BMC Infect Dis. 2008;8(1):99.

12. Ayu SM, Lai LR, Chan YF, Hatim A, Hairi NN, Ayob A, et al. Seroprevalence survey of Chikungunya virus in Bagan Panchor, Malaysia. Am J Trop Med Hyg. 2010;83(6):1245–8.

13. Sergon K, Yahaya AA, Brown J, Bedja SA, Mlindasse M, Agata N, et al. Seroprevalence of Chikungunya virus infection on Grande Comore Island, union of the Comoros, 2005. Am J Trop Med Hyg. 2007;76(6):1189–93.
